# Towards understanding aromatase inhibitory activity via QSAR modeling

**DOI:** 10.17179/excli2018-1417

**Published:** 2018-07-20

**Authors:** Watshara Shoombuatong, Nalini Schaduangrat, Chanin Nantasenamat

**Affiliations:** 1 Center of Data Mining and Biomedical Informatics, Faculty of Medical Technology, Mahidol University, Bangkok 10700, Thailand

**Keywords:** aromatase, aromatase inhibitors, breast cancer, estrogen, QSAR, structure-activity relationship, data mining

## Abstract

Aromatase is a rate-limiting enzyme for estrogen biosynthesis that is overproduced in breast cancer tissue. To block the growth of breast tumors, aromatase inhibitors (AIs) are employed to bind and inhibit aromatase in order to lower the amount of estrogen produced in the body. Although a number of synthetic aromatase inhibitors have been released for clinical use in the treatment of hormone-receptor positive breast cancer, these inhibitors may lead to undesirable side effects (e.g. increased rash, diarrhea and vomiting; effects on the bone, brain and heart) and therefore, the search for novel AIs continues. Over the past decades, there has been an intense effort in employing medicinal chemistry and quantitative structure-activity relationship (QSAR) to shed light on the mechanistic basis of aromatase inhibition. To the best of our knowledge, this article constitutes the first comprehensive review of all QSAR studies of both steroidal and non-steroidal AIs that have been published in the field. Herein, we summarize the experimental setup of these studies as well as summarizing the key features that are pertinent for robust aromatase inhibition.

## Introduction

Breast cancer is one of the leading causes of death worldwide, with a greater prevalence in developed countries and a rapidly growing health concern in developing countries. It is also the most frequently occurring cancer found in women with an estimated 1.5 million new cases resulting in 570,000 deaths in 2015 (WHO, 2015[99]). In addition, the prevalence of breast cancer in Asia is the highest among the world population (59 % of world population), out of which new cases account for 39 % with 44 % of cases resulting in deaths. In comparison, the prevalence of breast cancer in the continents of North America and Africa represent 5 % and 15 % of the world statistics, respectively (American Cancer Society, 2015[5]). 

**Figure 1:**
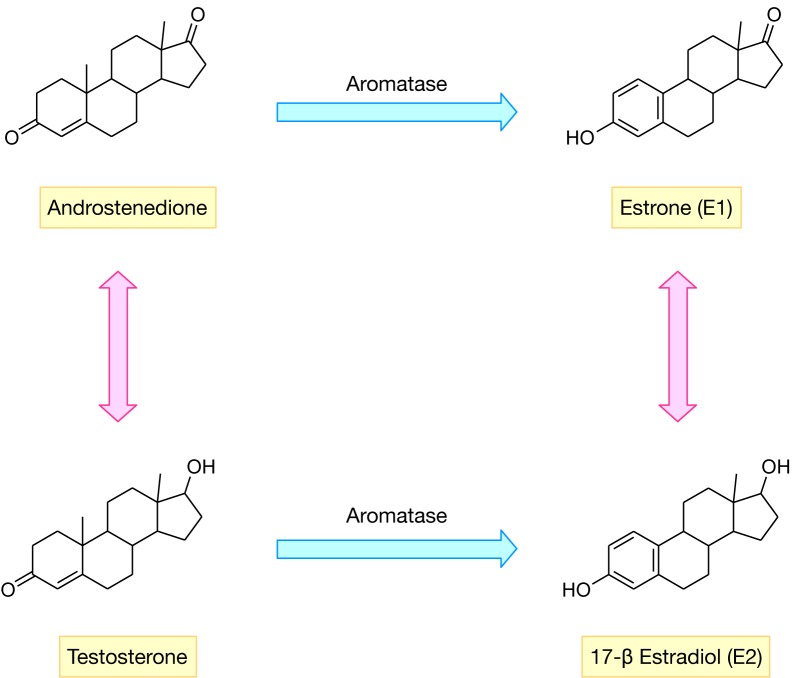
Summary of estrogen biosynthesis pathway as mediated by aromatase.

Estrogen is the primary female sex hormone that acts as a double-edged sword where on one side it regulates important physiological functions for sustaining life (i.e. regulating the menstrual cycle, modulating bone density, maintenance of vessels and skin etc.) while on the other side, it is implicated in the development of breast cancer. Estrogen biosynthesis is catalyzed by aromatase, which converts androstenedione, a 19-carbon (C_19_) steroid hormone, to estrone (E1) via a three-step A-ring aromatization. Aromatase also catalyzes the oxidation of testosterone, which is also then converted to estradiol (E2) (Ahmad and Shagufta, 2015[3]) (Figure 1(Fig. 1)). 

A common treatment for early-stage, hormone-sensitive breast cancer is surgery followed by radiotherapy. Furthermore, adjuvant endocrine therapy is given with or without chemotherapy depending on the tumor stage. In pre-menopausal women, most of the estrogen are made in the ovaries with the uptake of androstenedione from the circulation (Nelson and Bulun, 2001[66]). Ovaries can convert androstenedione to estrone via the catalytic activity of aromatase, which is then transported to breast cells. However, in post-menopausal women, the main site of estrogen production are the breasts. As for the latter, the level of estrogens produced in the breast are comparable to that produced in the ovaries by pre-menopausal women, which is four to six times higher than those found in serum. 

Approximately 60 % of pre-menopausal and 75 % of post-menopausal cancers are hormone-dependent (Russo et al., 2003[79]), implying that endogenous estrogens are essentially required for proliferation. Many drugs that are used for the treatment of estrogen receptor-positive breast cancer are mechanistically based on the interference of either the estrogen production or the estrogen action.

Aromatase, also known as estrogen synthase or CYP19A1, is part of the cytochrome P450 family. It is consisting of 503 amino-acid residues spanning twelve *α*-helices and ten *β*-strands, inside which a heme cofactor is coordinated by a cysteine residue at position 437 (Ghosh et al., 2009[33]). Aromatase is the major producer of estrogen in post-menopausal women and it catalyzes the rate-limiting step for converting androgens to estrogens (Simpson, 1994[89]). As aromatase catalyzes the biosynthesis of estrogen from androgens, thus the inhibition of aromatase activity has become the standard treatment for hormone-de-pendent breast cancers in women (Eisen et al., 2008[30]). Aromatase is located in the plasma membrane of the endoplasmic reticulum of estrogen producing cells and plays a role in development, reproduction, sexual different-iation and behavior as well as in bone and lipid metabolism, brain functions and diseases such as breast and testicular tumors. Hence, in order to block the estrogen production, it is necessary to inhibit the aromatase enzyme that is responsible for its synthesis by using aromatase inhibitors (AIs). AIs constitute the front-line therapy for estrogen-dependent breast cancers. For this reason, inhibiting this terminal step in the estradiol biosynthesis pathway is considered to be a specific and therefore, a preferable strategy.

## Aromatase Inhibitors

**Figure 2:**
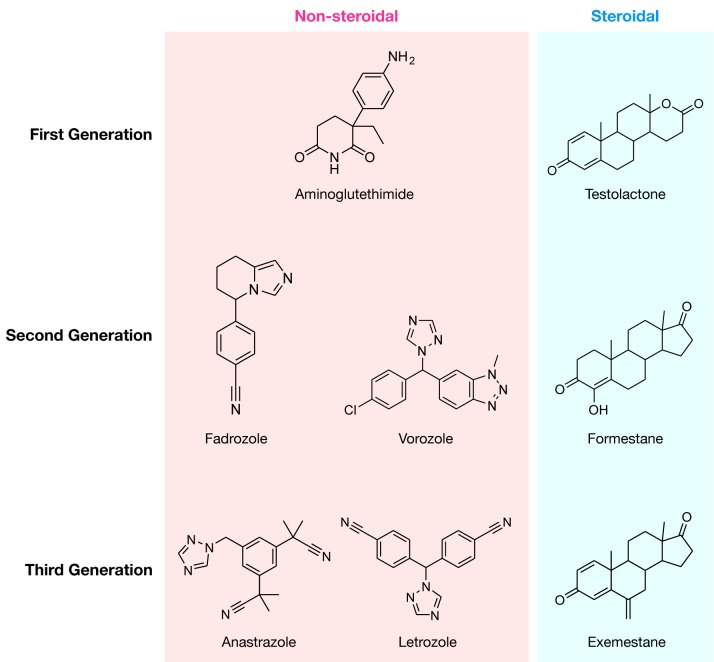
Chemical structures of the three generations of FDA-approved aromatase inhibitors.

To date, there are three generations of FDA-approved AIs available for inhibiting the activity of aromatase. The first-generation of AIs includes aminoglutethimide, which is marketed in the late 1970s (Santen et al., 1978[83], 1982[84]; Santen and Misbin, 1981[81]; Graves and Salhanick, 1979[35]) (Figure 2(Fig. 2)), a derivative of the sedative agent glutethimide that was initially introduced as an anticonvulsant. However, due to its adverse effects, such as high toxicity and lack of selectivity (Demers et al., 1987[26]; Hughes and Burley, 1970[41]), this AI was found to interfere with other CYP450 enzymes involved in cortisol and aldosterone biosynthesis (Santen et al., 1980[82]). Thus, aminoglutethimide was withdrawn from the market. In addition, testolactone was the first-generation steroidal AI that was used to treat advanced-stage breast cancers, albeit with weak potency (Avendaño and Menéndez, 2008[6]). Nevertheless, these first-generation AIs served as the prototype for future generations with an emphasis on developing more potent drugs with higher selectivity and reduced toxicity. Continuing on to the second-generation, fadrozole, which contains an imidazole group (Bonnefoi et al., 1996[13]), is more selective and potent than aminoglutethimide. Nevertheless, it still displayed effects on aldosterone, progesterone and corticosterone biosynthesis. Formestane (Brueggemeier et al., 2005[15]), a steroidal analogue, was the first selective AI to reach clinical trials in the 1990s. It was demonstrated to be effective and was well tolerated (Dowsett and Lloyd, 1990[27]). However, formestane exhibited poor oral bioactivity and had to be administered bi-weekly and thus, lost popularity with the discovery of the newer, more effective third-generation AIs (DrugBank, 2013[28]).

Finally, the third-generation of AIs, are comprised of triazole derivatives, anastrozole and letrozole and one steroidal analogue, exemestane (Dutta and Pant, 2008[29]). These AIs displayed improved efficacy and lower toxicity as compared with the estrogen antagonist, tamoxifen, in both early and advanced breast cancer (Thürlimann et al., 2004[95]). For this reason, the last generation of AIs has been recommended by the FDA as first-line drugs for the therapy of breast carcinoma. Anastrozole and letrozole, are non-steroidal derivatives and competitive inhibitors of androstenedione. Both contain a triazole group that can interact with the prosthetic heme group of aromatase. Exemestane is a steroidal analog of androstenedione thus, permanently binding to the enzyme and deactivating its catalytic activity (Coombes et al., 2007[19]). 

Initial attempts to clarify the interaction mechanism of aromatase and its inhibitors have relied on the use of homology-derived models (Loge et al., 2005[52]). Such studies have focused on clinically used AIs such as fadrozole, letrozole and exemestane as well as other natural products such as ligands, flavonoids and coumestrol (Karkola and Wähälä, 2009[43]; Paoletta et al., 2008[68]; Awasthi et al., 2015[7]; Worachartcheewan et al., 2014[103]; Nantasenamat et al., 2014[63]). 

Since the crystal structure of human placental aromatase has been solved by Ghosh et al. (2009[33]), the availability of structural details on the active site of aromatase helps in understanding of the binding characteristics of AIs as well as the evaluation of key reactions needed in the mechanism of aromatase. 

This has opened up a plethora of opportunities by enabling the understanding of the molecular basis for the specificity of the aromatase enzyme and its unique catalytic mechanisms, which is imperative for the development of the next-generation of AIs.

## Concepts of QSAR Modeling

Quantitative structure-activity relation-ship (QSAR) (Nantasenamat et al. 2009[59], 2010[60]) is a ligand-based approach that seeks to discern the mathematical relationship between chemical structures (i.e. as described by various types of molecular descriptors) and the investigated biological activity through the use of statistical and machine learning techniques.

Historically, the work of Cros (1863[22]), Crum Brown and Fraser (1868[23]) and especially that of Muir et al. (1949[54]) laid the foundations for the subsequent birth of QSAR as formally introduced by Hansch and Fujita (1964[37]) in their landmark work investigating substituent effects of various compounds against various biological activities (i.e. benzoic acids against mosquito larvae, phenols against gram-positive and gram-negative bacteria, phenyl ethyl phosphate insecticides against houseflies, thyroxine derivatives against rodents, diethyl-aminoethyl benzoates against guinea pigs and carcinogenic compounds against mice) by utilizing substituent constants as descriptors. Ever since, QSAR had been an integral part of computational drug discovery efforts as it had been utilized to probe the underlying mechanistic basis of various biological activities (Nantasenamat and Prachayasittikul, 2015[62]). Recently, Fujita and Winkler (2016[31]) had shared their perspectives on the two QSAR worlds consisting of (i) classical QSAR and (ii) modern QSAR.

The early years of *classical QSAR* entails investigation on the structure-activity rela-tionship of a congeneric set of compounds (i.e. compounds sharing a common chemical scaffold or chemotype) through the use of a few molecular descriptors. Classical QSAR methodology (Hansch et al., 1963[38]) assumes that the biological activity of investigated chemicals can be explained by simple and interpretable physicochemical properties. These physicochemical properties encode structural features that are considered to be statistically important and that can provide useful insights and understanding pertaining to the interaction being studied. Typically, classical QSAR models are built using partial least-squares (PLS) and multiple linear regression (MLR). It should be noted that this approach does not take into consideration the 3D structure of the receptor-ligand interaction. Thus, this had inspired the development of a 3D-QSAR technique by Cramer et al. (1988[21]) that essentially involves the alignment of a congeneric set of compounds (i.e. compounds sharing a common scaffold or chemotype) and followed by the computation of molecular fields (steric and electrostatic). Furthermore, modifications to the CoMFA concept known as comparative molecular similarity indices analysis (CoMSIA) was proposed by Klebe et al. (1994[45]) to extend CoMFA via the utilization of Gaussian potentials as the basis for calculating similarity and thus, expand its applicability (Kubinyi, 1997[46]). 

Over the years, advancements in computation has given rise to *modern QSAR* in which an extensive list of molecular descriptors as well as a wide range of machine learning algorithms can be applied in studying the structure-activity relationship of large sets of heterogeneous and chemically diverse set of compounds. On one end, modern QSAR makes it possible to harness the big data of bioactivity information accumulated over the years for model development while on the other end, the resulting models are often complex and not readily comprehensible to bench scientists. The need for simple and interpretable QSAR models along with best practices has been discussed in a recent book chapter (Shoombuatong et al., 2017[86]). Briefly, desirable characteristics of robust QSAR models have been set forth by the Organisation for Economic Co-operation and Development (OECD) as to encourage the utilization of QSAR models for regulatory purposes. These main OECD principles for the development of robust QSAR models are summarized in Table 1(Tab. 1).

**Figure 3:**
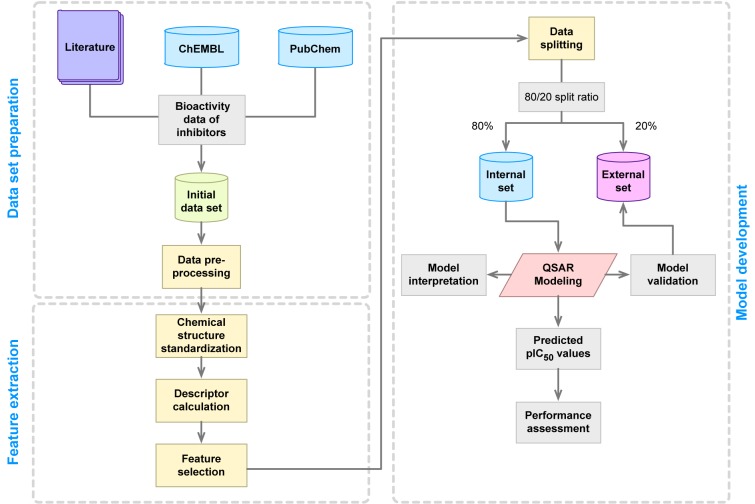
General workflow of QSAR model development.

The typical workflow for the development of QSAR models is depicted in Figure 3(Fig. 3). First, the QSAR modeling process starts by the compilation of a data set that entails collecting information pertaining to the compound name along with their SMILES notation, bioactivity values (e.g. IC_50_, EC_50_, K_i_, % activity, etc.) as well as reference to the original paper. Second, the data set is subjected to data pre-processing as to ensure the completeness of the data and that there are no missing information or misspellings. Third, chemical structures are drawn and subjected to structure standardization as to remove salts, ensure appropriate charge of functional moieties, select appropriate tautomeric structures, etc. Fourth, molecular descriptors are computed as to provide quantitative description of chemical structures and this is followed by feature selection as to remove useless and/or collinear variables. Fifth, the curated data set is employed for model construction via the use of machine learning algorithms and this entails data splitting, data balancing, data validation, model validation and performance assessment. Finally, the resulting model is subjected to scrutiny on the feature importance as to identify key features contributing to the origin of the biological activity. Summary and guidelines pertaining to the best practices for QSAR model development has been described previously (Tropsha, 2010[97]).

Since then, the breadth of available molecular descriptors have expanded to encompass a wide range of descriptors spanning one to several dimensions. Such descriptors may account for the general features of a molecule or may consider the fine details of a molecule down to its atomic constitution. A summary of common molecular descriptors (along with its description) used in QSAR models of AIs is provided in Table 2(Tab. 2) (References in Table 2: Cramer et al., 1988[21]; Kubinyi et al., 1998[47]; Polanski and Gieleciak, 2003[71]; Barigye et al., 2018[9]; Todeschini and Consonni, 2000[96]; Bak and Polanski, 2007[8]; Karelson et al., 1996[42]; Worachartcheewan et al., 2014[101]; Beger et al., 2004[11]).

### Machine learning

Machine learning is an implementation of artificial intelligence in which computers can automatically learn from data sets by extracting important patterns and making decisions or predictions. A summary of common machine learning algorithms that are used for QSAR modeling along with their strengths and weaknesses are provided in Table 3(Tab. 3). 

The concepts and in-depth treatment of machine learning is beyond the scope of this review and readers are directed to a previous comprehensive treatment of the topic (Shoombuatong et al., 2017[86]; van Westen et al., 2011[98]). Herein, we cover common machine learning algorithm that have been used in the study of AI activity.

The simplest learning algorithm is multiple linear regression (MLR) (Aiken et al., 2003[4]), which is an extension of the simple linear regression and is used to explain the relationship between a series of features, *X*=(*x_1_, x_2_, x_2_,…, x_N_*), and output values, *Y*=(*y_1_, y_2_, y_2_,…, y_N_*), as follows:


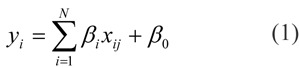


where *y_i_* is the output value, *x_ij_* is represented a data for *i^th^* compound and *j^th^* descriptor of *i^th^* compound and *β_i_* is the coefficient parameter.

*Partial least squares regression (PLS).* This method is a well-known method for constructing predictive models when features or descriptors have inter-correlated latent variables (Helland, 1988[39]; Helland, 2001[40]). PLS is closely related to principal component analysis (PCA) that consists of matrix decomposition into a matrix of eigenvectors and a matrix of its loadings factors. Given a dataset *X^N^*^x^*^M^* with N rows and M columns, the general approach can be written as follows: 


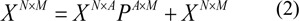


This is equivalent to a reduction of an *M*-dimensional variable space to an *A*-dimensional space. The variables in dimension *A* are also called latent variables. 

The matrix T contains orthogonal column vectors, also called score vectors, that represents the latent variables.

*Artificial neural network (ANN).* This method is a computation-based method inspired by networks of biological neurons in the human brain (Puri et al., 2016[74]). Basically, there are 3 different layers in the architecture of ANN: input layer (the input (*X*) is fed into the model through this layer), hidden layers (in general, there can be more than one hidden layers which utilizes some method to operate* X *and deliver to an output layer) and output layer (the data after processing is made available at the output layer). 

*Support vector machine (SVM).* This statistical learning approach is based on the principles of structure-risk minimization and kernel method as proposed by Cortes and Vapnik (1995[20]), which are used to construct a maximum-margin-separating hyperplane. The main advantage of SVM model is to seek the best compromise between the computational cost and the prediction error as to obtain the optimum generalization ability. SVM can be categorized as support vector classification (SVC) and support vector regression (SVR) (Cortes and Vapnik, 1995[20]; Smola and Schölkopf, 2004[90]). The principle idea of this method is to transform an input space with *m*-dimensional vector into a feature space with *n*-dimensional vector where* m < n*, and select a separating hyperplane giving the largest distance between the two classes.

*Decision tree (DT).* This machine learning technique is used for finding and describing a dataset (*X,Y*) with tree representation or structure (Safavian and Landgrebe, 1991[80]). The tree is composed of a root node, a set of internal nodes, and a set of terminal nodes (leaves). This method is one of well-known built-in feature selector. The main purpose of using DT is to achieve a more concise and transparency of the model to identify the relationship between *X* and *Y* variables. 

*Random forest (RF).* This ensemble learning method essentially integrates many classification and regression trees (CART). Breiman (2001[14]) developed the RF method by growing many weak decision trees for enhancing the prediction performance of CART. The last decade has witnessed the significant achievement of RF model in applications of drug developments and related works (Win et al., 2017[100]; Worachartcheewan et al., 2015[102]; Pratiwi et al., 2017[73]; Simeon et al., 2016[87]; Phanus-umporn et al., 2018[69]; Suvannang et al., 2018[94]). RF model takes advantage of two efficient machine learning techniques: bagging and random feature selection.

## QSAR Models of Aromatase Inhibitory Activity

The utilization of QSAR in aromatase research has only scratched the surface of the possible benefits that can be attained. Several classes of aromatase inhibitors have been created with only a few notable classes that have made it to the pre-clinical and clinical testing. 

Thus, it is worthwhile to elucidate the physicochemical profiles of effective aromatase inhibitors in comparison with ineffective ones as such knowledge can aid in the optimization of existing compound classes or development of novel classes from available scaffolds and functional group fragments. Particularly, questions such as “What molecular descriptors are crucial for highly potent com-pound? How big should a potent aromatase inhibitor be? Which functional groups are most commonly found in potent compounds?” could be answered through QSAR efforts. 

In 1997, Lipinski published a landmark paper on the Rule of 5 (Lipinski et al., 2001[51]), which has been widely used in the pharmaceutical industries as general guidelines for drug development efforts. The Rule of 5 considers ADMET issues that are critical towards the success of the identified compounds of interest as it may help reduce pre-clinical and clinical failures. A similar approach may be applied to the aromatase system where several Rules may be developed for the identification of potent aromatase inhibitors. 

The earliest QSAR study performed for AIs was published in 1994 by Nagy et al. (1994[58]) whereby MLR analysis was conducted on models built with 5 quantum chemical descriptors for 24 compounds assessed by LOO-CV procedure. From the results obtained, the authors were able to discover 2 candidate AIs for further pharmacophore studies. 

**Figure 4:**
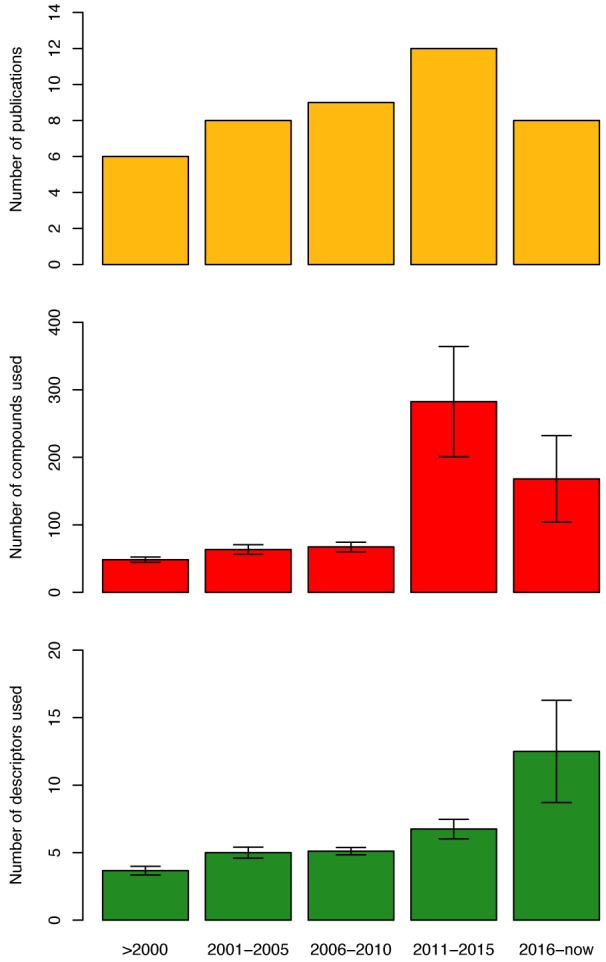
Overview on the number of publications (top), number of compounds (middle) and the number of descriptors (bottom) extracted from articles describing QSAR models of AIs.

Furthermore, as can be seen from Figure 4(Fig. 4) (top-left), in the years from 1994-2000 only five additional articles (Oprea and García, 1996[67]; Recanatini, 1996[75]; Sulea et al., 1997[92]; Recanatini and Cavalli, 1998[76]; Cavalli et al., 2000[18]) on QSAR of AIs were published, which utilized mainly molecular field descriptors and LOO-CV. For example, Cavalli et al. (2000[18]) quantitatively compared 3D-QSAR models of the cytochrome P450 active site via CoMFA modeling and homology modeling techniques. Once models were built, two non-steroidal AIs were docked into each model and the resulting interaction energies were recorded. The authors noted that although each technique had its drawbacks, both could be used together as a mutual validation technique for ligand-based and target-based 3D models of ligand-target interactions. 

In addition, Sulea et al. (1997[92]) described van der Waals envelopes as a steric potential field in a 3D-QSAR CoMFA based modeling of ligand-receptor interactions that was performed on 78 steroidal AIs and evaluated LOO-CV procedure. The authors were able to prove that the van der Waals envelopes intersection volumes (INVOL) could be used as an alternative replacement for the more commonly used Lennard-Jones 6-12 potential for the identification of relevant features governing biological activities within CoMFA and 3D-QSAR based models.

Similarly, Oprea and Garcia (1996[67]) analyzed the data of 50 steroidal AIs using CoMFA models coupled with chemometric based Generating Optimal Linear PLS Estimation (GOLPE) models validated using both the LOO-CV and the external test procedures. The authors concluded that using CoMFA, differences in aromatase inhibition among the C6-substituted steroids were shown to be consistent with known, potent inhibitors of aro-matase, included in the model. In addition, when direct alignment comparisons were made, these compounds exhibited distinct features that overlapped with the steric and electrostatic fields obtained in the CoMFA model. 

Over the course of the next five years (2001-2005) (Gironés and Carbó-Dorca, 2002[34]; Beger and Wilkes, 2002[12]; Beger et al., 2001[10]; Polanski and Gieleciak, 2003[71]; Beger et al., 2004[11]; Leonetti et al., 2004[50]; Cavalli et al., 2005[17]), it can be seen that studies employed higher number of descriptors as well as made use of more descriptor types (e.g. molecular fields, spectral, molecular surface and quantum chemical) were observed in seven publications where the only CV method applied on the datasets was the LOO-CV (Figure 4(Fig. 4)). Beger et al. (2004[11]) developed a technique which was similar to QSAR modeling, which they called the minimum deviation of structurally assigned spectra analysis (MiDSASA). This method was based on minimum chemical shift differences on substructure fragments instead of relying on substructure fragments as a whole for model production as is typical in SAR modeling. The authors used this MiDSASA template on 50 steroids binding the aromatase enzyme based on the average activity of the four nearest compounds, resulting in a correlation of 0.71. The authors further suggested that models made using the minimum deviation concept can be applied to other chemoinformatic data analyses such as metabolite concentrations in metabolic pathways for metabolomics research. 

In addition, Beger et al. (2001[10]) built quantitative spectroscopic data-activity relationship (QSDAR) models for 50 steroidal AIs developed based on data collected via simulated ^13^C nuclear magnetic resonance (NMR). The models were based on comparative spectral analysis (CoSA) and comparative structurally assigned spectral analysis (CoSASA). From the PLS analysis, the CoSA models exhibited *R^2^* of 0.78 and *Q^2^* of 0.71 while the CoSASA based models provided *R^2^* of 0.75 and* Q^2^* of 0.66. 

Similarly, Polanski and Gieleciak (2003[71]) used CoMSA to analyse the 3D-QSAR models built for 50 steroidal AIs. The authors aimed to predict regions that are important for the binding activity of the ligand with the enzyme. Using uniformative variable elimination as coupled to partial least squares (UVE-PLS) or modified iterative UVE procedure (IVE-PLS), the authors were able to determine that the 3D-QSAR models generated (*Q^2^* = 0.96) outperformed those reported at the time using CoMFA, CoSA or CoSASA.

Furthermore, the number of articles on QSAR of AIs were seen to increase rapidly for the years 2006-2010 (Figure 4(Fig. 4)) with the publication of ten articles in the time period (Bak and Polanski, 2007[8]; Nagar et al., 2008[55]; Castellano et al., 2008[16]; Mittal et al., 2009[53]; Gueto et al., 2009[36]; Nagar and Saha, 2010[57][56]; Roy and Roy, 2010[77][78]; Dai et al., 2010[24]). Most of the QSAR models in this time frame were built utilizing physicochemical descriptors as compared to other techniques in the previous years. 

**Figure 5:**
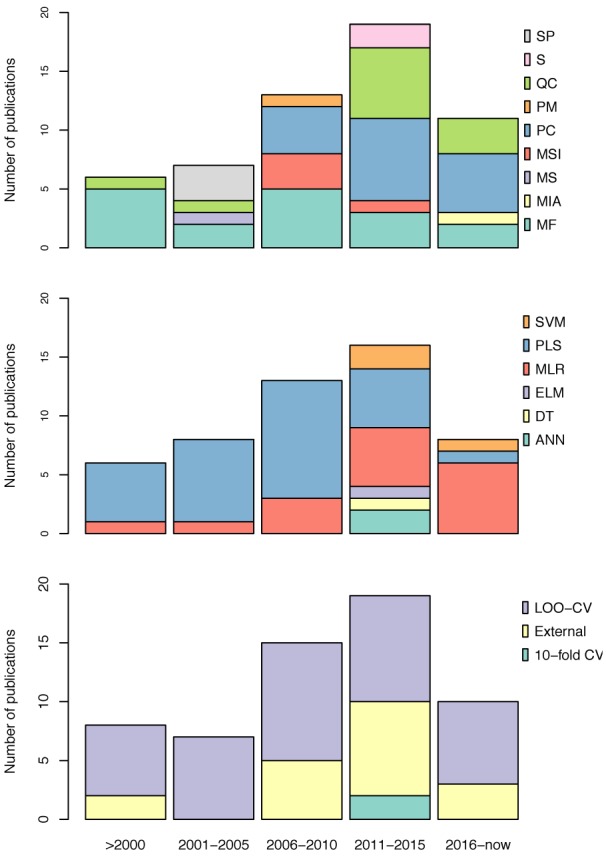
Overview of the types of descriptors (top), machine learning algorithms (middle) and validation methods (bottom) extracted from articles describing QSAR models of AIs. (Abbreviations: SP, S, QC, PM, PC, MSI, MS, MIA and MF represents spectral, SMILES, quantum chemical, pharmacophore mapping, physicochemical, molecular similarity indice, molecular surface, multivariate image analysis and molecular field, respectively. SVM, PLS, MLR, ELM, DT and ANN represents support vector machine, partial least square, multiple linear regression, efficient linear model, decision tree and artificial neural network, respectively. LOO-CV, external and 10-fold CV represents leave-one-out cross-validation, external test and 10-fold cross-validation, respectively)

Additionally, the validation methods for AIs QSAR publications in the abovementioned years were tied between LOO-CV only and LOO-CV in conjunction with external validations (Figure 5(Fig. 5)). For example, Bak and Polanski (2007[8]) conducted a 4D-QSAR study based on the self-organizing map (SOM), which is an unsupervised method based on the Kohonen neural network coupled with the IVE-PLS analysis. The use of this combined 4D-QSAR and IVE-PLS method provided a very stable and predictive modeling technique. The method enabled the authors to identify molecular motifs contributing to the aromatase enzyme binding activity. Gueto et al. (2009[36]) employed structure-based drug design approach using receptor-independent CoMFA maps that were generated from LeapFrog calculations. 

A robust model as verified by the bootstrapping method produced statistically significant results via cross-validated analysis, which consisted of 45 and 10 molecules in the training and test sets, respectively. Using this model, the authors were able to predict the activity of novel AI molecules which had more potency than previously reported compounds. Roy and Roy (2010[77]) performed a 3D-QSAR study on a diverse set of compounds using the crystal structure of aromatase whereby the dataset was divided into training (n=87) and testing (n=29) set by clustering techniques. All the QSAR models were subjected to multiple validation methods such as internal validation, external validation and Y-randomization. The authors concluded that in order to exhibit ideal aromatase inhibitory activity, the compound should contain at least one or two hydrogen bond acceptor groups (such as NO_2_ and CN) with optimal hydrophobicity. 

Additionally, the increase in popularity of QSAR models for predicting AIs was greatly observed in 2011-2015 (Narayana et al., 2012[65]; Kishore et al., 2013[44]; Nantasenamat et al., 2013[61][64], 2014[63]; Dai et al., 2014[25]; Xie et al., 2014[104]; Worachartcheewan et al., 2014[101][103]; Shoombuatong et al., 2015[85]; Awasthi et al., 2015[7]; Xie et al., 2015[105]; Kumar et al., 2016[48]) whereby the number of publications increased to thirteen, with an even more dramatic rise in the number of compounds used for calculating descriptors using LOO-CV and external validation (Figure 5(Fig. 5)). Worachartcheewan et al. (2014[103]) investigated the QSAR of coumarins as potential AIs using 7 quantum chemical descriptors. MLR was used for the analysis of models, which were shown to achieve good predictive performance as verified by LOO-CV affording *Q^2^* of 0.9239 and RMSE_CV_ of 0.1304 while an external validation confirmed its robustness with *Q^2^_Ext_* of 0.7268 and RMSE_Ext_ of 0.2927. 

Moreover, Nantasenamat et al. (2013[64]) explored a set of 54 letrozole analogs as AIs in a QSAR study using MLR, ANN and SVM methods. The QSAR model was developed using a set of descriptors giving rise to important physicochemical properties (i.e. number of rings, ALogP and HOMO-LUMO) which were further used for predicting AI activity. The authors observed a strong correlation among the predicted pIC_50_ values with their experimental values, displaying correlation coefficient *Q^2^* values in the range of 0.72-0.83 while the external test set (*Q^2^_Ext_*) afforded values in the range of 0.65-0.66. Furthermore, Worachartcheewan et al. (2014[101]) employed the bioactivity data on pIC_50_ of 973 AIs for constructing QSAR models using CORelation And Logic (CORAL) software (http://www.insilico.eu/coral) where the molecular structures are represented by their simplified molecular input line entry system (SMILES) notation and thus eliminating the need to geometrically optimize molecular structures. The Monte Carlo optimization of correlation was used for predicting the aromatase inhibitory activity. Results obtained from rigorous dataset splits and CV techniques indicated that models were reliable with *R^2^* and *Q^2^* in ranges of 0.6271-0.7083 and 0.6218-0.7024, respectively. Similarly, Nantasenamat et al. (2014[63]) conducted the first large-scale QSAR study on a non-redundant set of 63 flavonoids using MLR, ANN, SVM and DT methods. The models obtained showed good predictive performance with *Q* values in the range of 0.8014-0.9870 and 0.8966-0.9943 evaluated by LOO-CV and external test, respectively. Furthermore, in another study conducted by our group Shoombuatong et al. (2015[85]), proposed the simple and interpretable efficient linear method (ELM) for constructing a highly predictive QSAR model. The results indicated that a robust performance was achieved using the ELM method with 10-fold CV MCC values of 0.64 and 0.56 for steroidal and non-steroidal AIs, respectively. In addition, Xie et al. (2014[104]) constructed 3D QSAR models in order to elucidate the steroidal AIs with lower side effects using CoMFA and CoMSIA methods. The models produced were reliable and predictive good statistical results for CoMFA: *Q^2^* = 0.636, *R^2^* = 0.988, *Q^2^_Ext_* = 0.658 and CoMSIA: *Q^2^* = 0.843, *R^2^* = 0.989, *Q^2^_Ext_* = 0.601. 

The current trend (2016-2018; Figure 4(Fig. 4)) shows that eight articles (Song et al., 2016[91]; Ghodsi and Hemmateenejad, 2016[32]; Adhikari et al., 2017[1]; Prachayasittikul et al., 2017[72]; Pingaew et al., 2018[70]; Lee and Barron, 2018[49]; Barigye et al., 2018[9]) have already been published in comparison to a total of 13 publications for the previous 5 years. Thus, it is promising that the number of publication regarding AIs utilizing QSAR models for prediction will continue to grow. 

To further aid in that growth process, the number of compounds used as the data set has seen a steady rise with the number of descriptors for generating QSAR models saw a dramatic increase as compared to previous years. As for the types of descriptors, the trend has moved towards modern QSAR with the utilization of physicochemical properties and quantum chemical structures to build the models. In addition, the main validation techniques remain the same as previous years whereby LOO-CV and external validation were mainly used. Ghodsi and Hemmateenejad (2016[32]) conducted QSAR studies on a series of diarylalkylimidazole and diarylalkyltriazole derivatives previously evaluated as being potent AIs using 870 quantum chemical descriptors (such as dipole moment and energies of HOMO and LUMO orbitals, hydration energies, and lipophilicity) that were analyzed using MLR. The models were validated with the LOO-CV and the authors concluded that lipophilicity was an important factor for the strong binding to aromatase. In addition, the HOMO orbital shape and its imidazole ring distribution was also considered as important. More recently, Adhikari et al. (2017[1]) performed QSAR studies using various techniques (2D-QSAR, 3D-QSAR and HQSAR) on 67 non-steroidal letrozole-based analogs with promising AI activity. Stepwise multiple linear regression (SMLR) was used to build the models after which, the models were validated with the LOO for internal validation. The results from the 2D-QSAR study suggested the importance of the nitrogen atoms in their electrotopological state thereby inferring that their orientation may modulate the inhibition. The authors noted that these results were further validated with the 3D-QSAR analysis while the HQSAR model inferred the importance of the *p*-cyanophenyl moiety in regulating AI. Additionally, Lee and Barron (2018[49]) conducted 3D-QSAR studies on the bioactivity (IC_50_) of 124 compounds exhibiting AI activity (steroidal and heterocyclic). Multiple linear regress-ion combined with genetic algorithm (GA-MLR) was used to build the models which was then validated via the LOO and external validation methods. Furthermore, Prachayasittikul et al. (2017[72]) investigated the aromatase inhibitory potency of a series of 2-amino (chloro)-3-chloro-1,4-naphthoquinone derivatives by constructing QSAR models using the IC_50_ values. The models were evaluated based on MLR and LOO-CV which indicated good predictive performance (*Q^2^* = 0.9783 and RMSE_CV_ = 0.0748) of the constructed model. Therefore, 1,4-naphthoquinone derivatives can be seen as promising compounds needed further evaluations as AIs. The most recent article published by Barigye et al. (2018[9]) reported the first practical application of Discrete Fourier Transformation (DFT) based Multiple Image Analysis (MAI) derived 2D-QSAR model for the classification of an aforementioned set of 973 novel AIs as compiled from the literature (Nantasenamat et al., 2013[61]).

## Insights from QSAR Models

Model interpretation is the process by which the underlying features contributing the most to the investigated biological activity are deduced as to help guide the design of novel and robust drugs. The interpretability of a QSAR model is contingent upon the types of descriptors and machine learning algorithms used. As summarized in Table 4(Tab. 4) (References in Table 4: Nagy et al., 1994[58]; Recanatini, 1996[75]; Oprea and García, 1996[67]; Sulea et al., 1997[92]; Recanatini and Cavalli, 1998[76]; Cavalli et al., 2000[18]; Beger et al., 2001[10]; Gironés and Carbó-Dorca, 2002[34]; Beger and Wilkes, 2002[12]; Polanski and Gieleciak, 2003[71]; Leonetti et al., 2004[50]; Beger et al., 2004[11]; Cavalli et al., 2005[17]; Bak and Polanski, 2007[8]; Castellano et al., 2008[16]; Nagar et al., 2008[55]; Mittal et al., 2009[53]; Gueto et al., 2009[36]; Dai et al., 2010[24]; Roy and Roy, 2010[78]; Roy and Roy, 2010[77]; Nagar and Saha, 2010[57]; Nagar and Saha, 2010[56]; Narayana et al., 2012[65]; Nantasenamat et al., 2013[64]; Nantasenamat et al., 2013[61]; Kishore et al., 2013[44]; Worachartcheewan et al., 2014[103]; Worachartcheewan et al., 2014[101]; Nantasenamat et al., 2014[63]; Dai et al., 2014[25]; Awasthi et al., 2015[7]; Xie et al., 2015[105]; Shoombuatong et al., 2015[85]; Xie et al., 2014[102]; Kumar et al., 2016[48]; Ghodsi and Hemmateenejad, 2016[32]; Song et al., 2016[91]; Prachayasittikul et al., 2017[72]; Adhikari et al., 2017[1]; Lee and Barron, 2018[49]; Pingaew et al., 2018[70]; Barigye et al., 2018[9]), it can be observed that prior to 2010, MLR and PLS models, also known as white-box approaches, were the most popular and yet simple learning algorithms used for QSAR modeling of AIs. 

Although these two models are interpretable but they did not perform well on highly complexed data. On the other hand, a black-box approach like ANN and SVM can provide higher accuracy in the same case but they cannot provide details pertaining to how the factors exert its influence on the biological activity of investigated compounds. Analysis of key features for aromatase inhibition from selected QSAR works employing descriptors pertaining to quantum chemical and physicochemical properties are performed hereafter (Table 5(Tab. 5)). Nantasenamat et al. (2013[61]) performed a large-scale QSAR modeling of a set of steroidal and non-steroidal AIs and revealed that the most important features from PCA analysis were found to be nHAcc, TPSA and LUMO for non-steroidal and Q_m_, TPSA and nHAcc and ALogP for steroidal AIs. In addition, fragment analysis provided complementary insights by suggesting that the presence of the azole ring in non-steroidal inhibitors (i.e. known to coordinate with the heme iron) and the presence of carbonyl group in the C3 position of steroidal inhibitors were important for aromatase inhibition.

In addition, using the same set of data, Shoombuatong et al. (2015[85]) used the ELM model to deduce the most important features associated with AI. It was observed that the top four most informative descriptors for the steroidal dataset were C-025 (atom centered fragments; R--CR--R), ESpm14u and ESpm13r (connectivity or bonding between atoms) and MATS6p (involved in polarizability of molecules).

As for the non-steroidal dataset, the most important feature was determined to be molecular graph, polarizability and electronegativity of the compound. Therefore, the authors concluded that the polarizability of the compounds along with a suitable shape may be the determining factors needed for both types of AIs for reaching its intended target. Additionally, Worachartcheewan et al. (2014[101]) conducted a large-scale study on AIs using SMILES-based descriptors and discovered that the most notable features were the presence of cyclic rings (i.e. found in steroidal inhibitors) and the presence in the molecular structure of oxygen atoms together with double bonds that are disconnected in the structure (++++O---B2==) (i.e. analogous to the ketone groups present in the natural substrate, androstenedione) are important in increasing aromatase inhibitory activity. Furthermore, Ghodsi and Hemmateenejad (2016[32]) conducted QSAR on AIs based on long-chained diarylalkylimidazole and diarylalkyltriazole (non-steroidal) molecule skeletons in which they determined important features to include geometrical distances of N and N atoms as well as that of O and O atoms (i.e. nitrogen atoms of azole rings as well as oxygen atoms from steroidal ketones), length of the bridge carbon chain (i.e. methylene spacer separating the azole ring and the phenol ring), number of triple bonds (i.e. triple bond in the nitrile or CN that is an integral part of FDA-approved AIs), HOMO energy (i.e. localization of HOMO orbital predominantly in the imidazolyl ring), etc. Furthermore, Nantasenamat et al. (2014[63]) studied flavonoids with aromatase inhibitory activity, and found that active compounds were found to exhibit smaller size, higher degree of rigidity, lower polarity and charge distribution, and afforded lower electron-withdrawing tendency and higher chemical reactivity than those of the inactive class.

As for the analysis of 3D-QSAR models utilizing descriptors based on molecular fields, Castellano et al. (2008[16]) revealed that the aligned molecules showed the presence of three major regions in which two were pertinent for aromatase inhibition (i.e. one important region afforded both electrostatic and hydrogen bonds while the second important region was occupied by the characteristic azole moiety) whereas the other region was not important for the activity. Adhikari et al. (2017[1]) performed an extensive study employing a wide range of QSAR models including 2D and 3D QSAR as well as molecular docking to also confirm the importance of the electrostatic property of the nitrogen-containing azole moiety, *p*-cyanophenyl moiety, *p*-nitrophenyl, hydro-phobicity as well as the appropriate size and shape of AIs were crucial for aromatase inhibitory activity. Xie et al. (2015[105]) performed both CoMFA and CoMSIA studies and both studies further supported the importance of bulky steric groups as well as the importance of electrostatic properties pertaining to the presence of azole nitrogen atoms.

## Conclusion

In spite of extensive research (i.e. medicinal chemistry and QSAR work) in the quest of novel and potent aromatase inhibitors, there has been only a few review articles on the topic (Adhikari et al. 2017[2]; Yadav et al. 2015[106]). Briefly, Yadav et al. (2015[106]) carried out a review focusing on molecular modeling as well as QSAR of steroidal AIs whereas Adhikari et al. (2017[2]) based their review on QSAR studies of non-steroidal AIs. Herein, we have performed an extensive review on the mechanistic insights of pertinent features as derived from all previous QSAR models of both steroidal and non-steroidal AIs. Moreover, this review also summarized the experimental setup of all QSAR studies such that a comparative and holistic analysis could be deduced and used for providing a glimpse on the current state-of-the-art in the field as well as serving as the basis for planning future studies to further gain insights on aromatase inhibition. For example, it is anticipated that insights gained from QSAR models alone provides one aspect where it may be beneficial to also call upon complementary methodologies such as structure-based and systems-based approaches to facilitate and augment the ligand-based QSAR approach. In fact, there have been a few studies employing a multitude of ligand, structure and systems-based approaches in studying aromatase inhibition (Simeon et al. 2016[88]); Suvannang et al. 2011[93]) and future works along this line is expected to be of great benefit to the scientific community.

## Conflict of interests

The authors have declared that no competing interests exist.

## Acknowledgements

This work is supported by the Office of Higher Education Commission and the Thailand Research Fund (No. MRG6180226) and the New Researcher Grant (A31/2561) from Mahidol University. This work is also supported by the Center of Excellence on Medical Biotechnology (CEMB), S&T Postgraduate Education and Research Development Office (PERDO), Office of Higher Education Commission (OHEC), Thailand. Partial support from the annual budget grant (B.E. 2557-2559) of Mahidol University is also acknowledged.

## Figures and Tables

**Table 1:**
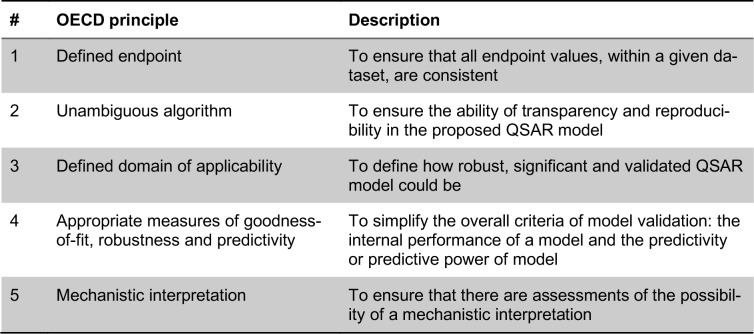
The OECD principle guidelines for developing and validating QSAR model.

**Table 2:**
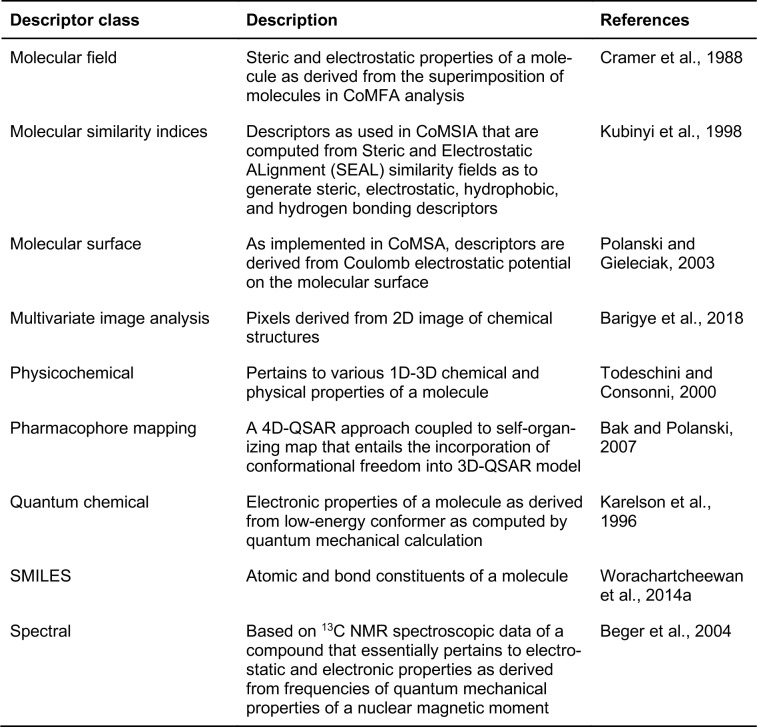
Summary of common classes of molecular descriptors.

**Table 3:**
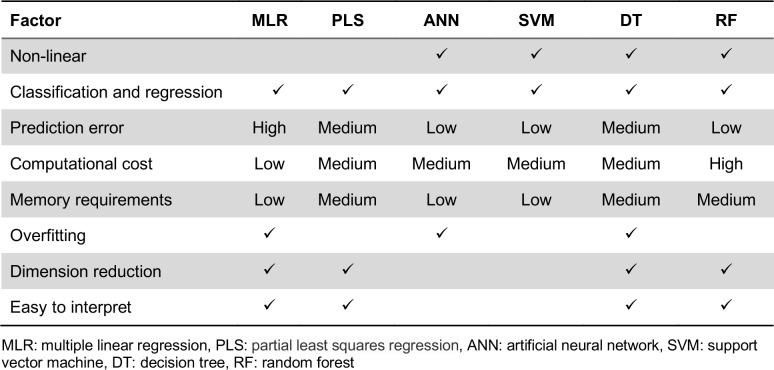
Summary of the strength and weakness of the machine-learning algorithms for performing QSAR modeling discussed in this review.

**Table 4:**
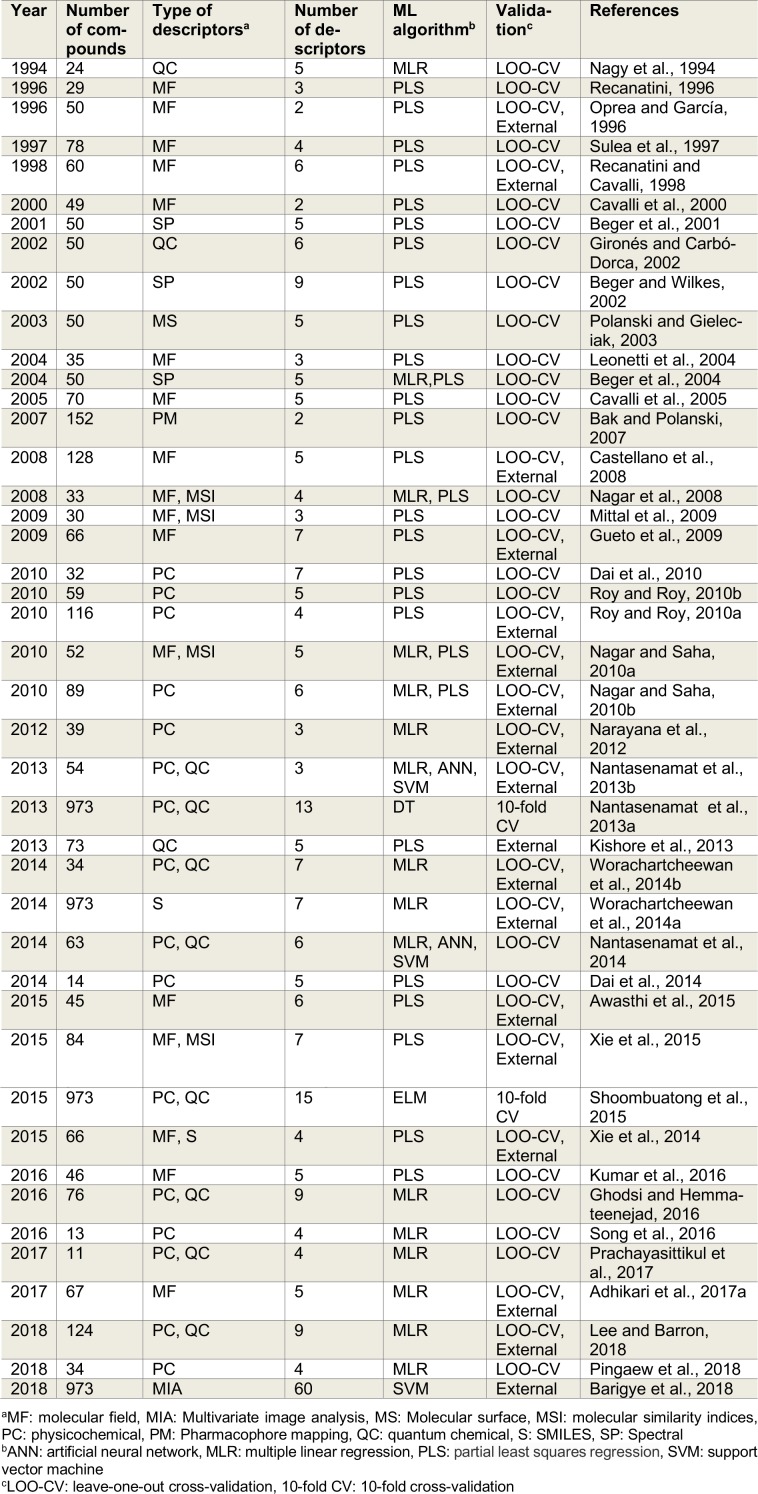
Summary of machine learning algorithm used in QSAR modeling for predicting and analyzing aromatase inhibitor.

**Table 5:**
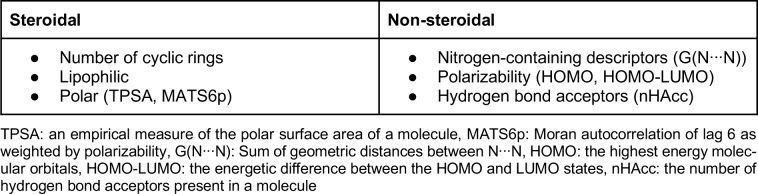
Summary of key features for aromatase inhibition as deduced from QSAR modeling. Example descriptors are shown in the parenthesis.
